# Mental Health Support Services in Universities: A Dyadic Exploration of Students’ and Counsellors’ Perspectives

**DOI:** 10.3390/healthcare14162606

**Published:** 2026-08-19

**Authors:** Nur Natasha Kamarudin, Puteri Fadzline Muhamad Tamyez, Wan Khairul Anuar Wan Abd Manan, Shishi Kumar Piaralal, Abdul Rahman bin S Senathirajah, Premala Devi Sivagurunathan, Poh Kiat Ng, Peng Qin, Rubentheran Sivagurunathan

**Affiliations:** 1 Faculty of Industrial Management, Universiti Malaysia Pahang Al-Sultan Abdullah, Gambang 26300, Malaysia; 2 Faculty of Business and Management, Open University Malaysia, Petaling Jaya 47301, Malaysia; 3 Faculty of Business and Communication, INTI International University, Persiaran Perdana BBN Putra Nilai, Nilai 71800, Malaysia; 4 Wagga Wagga Base Hospital, Wagga Wagga, NSW 2650, Australia; 5 Centre of Advanced Mechanical and Green Technology, Multimedia University, Jalan Ayer Keroh Lama, Bukit Beruang 75450, Malaysia; 6 Faculty of Liberal Arts, Shinawatra University, Sam Khok District, Pathum Thani 12160, Thailand

**Keywords:** mental health, psychological well-being, education, qualitative research, help-seeking behaviour, mental health support

## Abstract

**Background:** Mental health issues among university students are a growing global concern, including in Malaysia. This exploratory study aims to examine mental health support systems in Malaysian universities using a dyadic perspective, capturing both students’ and counsellors’ experiences. This addresses a key gap in the literature, which has largely relied on single-perspective accounts. **Methods:** A qualitative exploratory design was employed using purposive sampling. Semi-structured interviews were conducted with 15 students receiving mental health support and 10 university counsellors. The data were analysed using hybrid deductive–inductive thematic analysis. **Results:** Three themes emerged from the findings. The first theme showed that students sought support once distress affected their daily functioning, valuing trust, safety, and emotional guidance, while counsellors described a parallel process of assessing student needs and facilitating longer term recovery. The second theme identified barriers shared by both groups, namely limited awareness of services and stigma, alongside additional constraints reported only by counsellors, including staffing shortages, unclear referral pathways, and limited institutional support. The third theme centred on strategies for improvement, including early assessment, awareness campaigns, digital accessibility, resilience building, and stronger collaboration among students, counsellors, and university stakeholders. **Conclusions:** This study contributes to the literature by integrating three complementary theoretical perspectives to explain university students’ mental health support experiences, support networks, and help-seeking behaviour. The findings also provide practical guidance for university administrators, counsellors, and policymakers seeking to develop more accessible, proactive, and student-centred mental health support systems. As the study is based on qualitative, cross-sectional data, the findings should be interpreted as indicative rather than causal. Nonetheless, they suggest that strengthening such initiatives may contribute to more supportive and responsive mental health provision within higher education institutions.

## 1. Introduction

Mental health is more than the absence of mental health conditions. Rather, it is a state of mental well-being that enables people to cope with the stresses of life, realise their abilities, learn well and work well, and contribute to their communities [1]. Mental health has become a growing global concern, with Malaysia reflecting similar trends. The Malaysian Youth Mental Health Index 2023 reported that 60% of Malaysian youths experienced mild to severe depressive symptoms, while 10% reported suicidal ideation [2]. National data indicate that 2.3% of adults experience depression, while 7.9% of children aged 5–15 experience mental health problems [3]. This evidence warrants an immediate need for accessible, effective, and responsible mental health support systems within Malaysian universities.

In response, Malaysian universities have introduced a range of measures, from awareness campaigns and psychoeducation to more substantial campus support systems. Notable examples are Universiti Putra Malaysia’s screening and mindfulness workshops, University of Wollongong Malaysia’s Connectopia event series, Asia Pacific University’s clinical screening collaboration with The Pillars Malaysia, and Monash University Malaysia’s mental health first aid training and peer support groups. Demographic factors such as ethnicity, religion, and age have also been shown to influence stigma and help-seeking attitudes [4], suggesting that culturally tailored interventions are needed rather than conventional, one-size-fits-all approaches.

Despite these initiatives, mental health concerns remain prevalent, and comparatively little attention has focused on how support services are experienced, utilised, and delivered. Existing research has generally provided only a single perspective, either the student’s or the institution’s, and has identified contributing factors such as academic pressure, financial constraints, and the transition to university life [5,6,7] without examining how support itself is perceived and delivered by those on both sides of it.

This points to three related gaps in the literature. Firstly, existing studies have focused more extensively on the causes of mental health difficulties than on perceptions of and the effectiveness of available support services. Secondly, most studies have examined only students’ perspectives, overlooking counsellors as key service providers within the support system. Thirdly, limited research has explored the perspectives of both students who have used counselling services and the counsellors who provide them. This has resulted in an incomplete understanding of how support is perceived and delivered, particularly given the limited empirical evidence from Malaysian universities. Addressing this gap requires consideration of both perspectives, as an effective support system depends on alignment between students’ expectations and counsellors’ capabilities and practices.

To address this gap, the present study adopts a dyadic perspective by examining both service users and service providers. It is guided by an integrated multi-theoretical framework comprising Self-Determination Theory, Social Support Theory, and Help-Seeking Behaviour Theory. This dual perspective provides a more balanced and comprehensive understanding than a single-perspective design and contributes to the literature. Firstly, it makes a theoretical contribution by integrating three complementary theories to explain mental health support experiences, support networks, and help-seeking behaviour. Besides that, it offers a methodological contribution through the use of a novel qualitative dyadic design. Lastly, it provides a practical contribution by generating empirical, data-driven insights that may help universities, counsellors, and policymakers develop more responsive and student-centred mental health support systems.

Based on the identified gaps and theoretical considerations, this study seeks to answer the following three research questions:

RQ1: How do students who used counselling services and counsellors, experience and interpret mental health support services in Malaysian universities, and in what ways do their accounts converge or diverge?
RQ2: What barriers and challenges shape the utilisation and delivery of mental health support services in Malaysian universities, from the perspectives of students who used counselling services and counsellors?
RQ3: What strategies do students who used counselling services and counsellors propose to enhance mental health support services in Malaysian universities?

The structure of this paper is organised as follows. Section 2 reviews the literature on global perspectives on mental health and discusses the study’s multi-theoretical framework. Section 3 outlines the research design, including participant selection, data collection procedures, and data analysis techniques. Section 4 presents the thematic findings, while Section 5 discusses these findings in relation to the literature and relevant theoretical perspectives. Finally, Section 6 concludes the paper by outlining its theoretical and practical implications, limitations, and recommendations for future research.

## 2. Literature Review

### 2.1. Global Perspective on Mental Health Amongst Youths

Numerous studies across the globe have provided a comprehensive assessment of the mental health issues afflicting our societies. These studies have recurrently posited that mental health is a key threat impeding the development of the younger generation worldwide. In particular, mental health is escalating into an issue which is detrimental to the growth of the youths worldwide [8]. Despite growing awareness of this issue, structural and systemic barriers continue to restrict access to timely, high-quality clinical interventions and effective mental health support services [9]. This crisis is particularly pronounced in low- and middle-income countries. In these countries, pervasive stigma and limited mental health infrastructure further intensify the burden of common mental health conditions, such as anxiety and depression [10]. Evidence from 165 countries indicates that rising economic inequality is an important factor contributing to mental health difficulties. Greater income inequality has been associated with lower life satisfaction and increased emotional distress, particularly among young people and those from lower-income groups [11].

To bridge these gaps, emerging research emphasises that integrating digital solutions with traditional therapy is essential for scaling services and overcoming geographic disparities [9,12]. Integrated primary care platforms and school-based mental health promotion programmes have demonstrated promising outcomes in low- and middle-income countries. In particular, universal life-skill interventions have been found to improve well-being and reduce behavioural problems when implemented using high-quality methods [13]. Research has also shown that personalised support which can be understood by young people and professionals from different contexts should be provided, including marginalised and minoritized groups and communities [14].

Addressing these multifaceted challenges requires a shift toward cross-sectoral collaboration and increased investment to bridge the gap between burgeoning mental health literacy and the availability of evidence-based care [15,16]. Furthermore, effective and sustainable interventions to address the global burden of mental disorders in children and adolescents should address the social, cultural and economic challenge [17].

Transitioning towards a transformative early intervention paradigm for research and clinical care could significantly enhance mental health in young people and initiate a shift towards the prevention of severe mental disorders [18]. Moreover, shifting the focus toward real time data collection allows for closer monitoring, which is critical for timely, preventive action [19]. This transition toward a prevention-focused framework requires a wholesale policy transformation that prioritises frequent mental health tracking and the integration of multidisciplinary care models [20] where the school counsellor and health professionals work in tandem, as part of transformative priorities for long-term reform. Such an approach requires urgent and coordinated action across the health, education, social services, and justice sectors [21].

### 2.2. Multi-Theoretical Perspective

This study adopted three complementary theories, rather than a single dominant theory, to examine mental health support systems: Self-Determination Theory, Social Support Theory, and Help-Seeking Behaviour Theory. Each theory directly shaped this study’s research questions, interview protocol, coding framework, and interpretation of findings.

Self-Determination Theory explains individual motivation through the three basic psychological needs of autonomy, competence, and relatedness [22,23]. This theory informed RQ1’s focus on how students and counsellors experience mental health support, guided interview questions on trust, coping, and confidence building, and structured deductive coding of the autonomy, competence, and relatedness dimensions. In the findings, Self-Determination Theory accounted for students’ emphasis on trust and safety in the counselling relationship, and counsellors’ emphasis on building students’ coping capacity.

Social support-based theories collectively explain how emotional, instrumental, informational, and appraisal support from social networks buffer psychological distress [24,25]. This theory informed RQ1 and RQ3 by directing attention to the roles of counsellors, lecturers, peers, and other stakeholders. It also guided the interview questions on sources of support and informed the deductive coding of the four support dimensions. It accounted for findings on professional and peer support networks and stakeholder collaboration strategies.

Help-Seeking Behaviour Theory explains help-seeking through problem recognition, attitudes, perceived stigma, and service accessibility [7,26]. This theory directly informed RQ2’s focus on barriers to utilisation and delivery, guided interview questions on awareness, stigma, and access, and structured deductive coding of these four dimensions. It accounted for the bulk of the barrier and strategy findings including awareness gaps, stigma, and service accessibility.

Together, these three theories informed a coding framework encompassing the individual, relational, and behavioural dimensions of mental health support. Findings that did not align with the three theoretical perspectives, such as counsellor-reported staffing shortages and institutional constraints, were treated as boundaries of the framework. These findings are presented in Section 4.2 and revisited in the Section 5.

## 3. Research Methodology

### 3.1. Research Design

This study adopted an interpretivist research philosophy, based on the assumption that the social reality experienced is subjectively constructed through the meanings that humans attach to their lived experiences [27,28,29]. Because such subjectively constructed meaning is best accessed through in depth, context-sensitive inquiry rather than measurement, the study employed a qualitative exploratory design [30]. The dyadic approach enables comparison of the accounts of two interconnected parties [31,32]. The two parties in this case are students who used counselling services and counsellors, yielding a more complete and triangulated understanding of mental health services. This entire process surfaced points of convergence and divergence that a single-perspective design would overlook [33,34].

### 3.2. Participants and Sampling

The aim of this study was to explore mental health support services in Malaysian universities from the perspectives of students who used counselling services and counsellors. In line with this objective, a purposive sampling strategy was employed [35]. The basis of using purposive sampling was to identify individuals with relevant experience of the phenomenon [36], which was mental health support systems. Participants were recruited from several universities across Malaysia through email invitations. Eligible student participants were required to have used university counselling services at least once. However, a formal clinical diagnosis was not required for inclusion. All individuals who were approached agreed to participate, and no refusals or dropouts were recorded. As shown in Table 1, the final sample comprised 15 students who had used counselling services and 10 counsellors. The student participants covered students from different years of study to capture viewpoints from a diversity of student seniority. The counsellors had between seven and sixteen years of professional counselling experience, ensuring a range of work experience was covered. This diversity from both participant types enabled the study to capture a broad range of perspectives on mental health support services. The sample size was considered appropriate for the present study, as the analysis focused on identifying broad, overarching themes. Previous research has suggested that a sample of six interviews may be sufficient to generate meaningful themes and useful interpretations when the study aims to explore high-level patterns [37]. Consistent with recent guidance on thematic analysis, which cautions against relying on saturation as a sample size rationale [38], sample adequacy was instead assessed reflexively throughout data collection using the concept of information power [39]. Information power suggests that the more relevant information a sample provides for addressing the study aim, the fewer participants may be required. In light of this, this study’s recruitment continued until the accumulated data provided sufficiently rich and relevant insight into the study’s research questions across both student and counsellor perspectives.

### 3.3. Data Collection

Prior to data collection, ethical approval was obtained from the university review board. In this study, the COREQ checklist was used to ensure comprehensive and transparent reporting of the qualitative methodology, thereby enhancing the rigour and credibility of the findings [40]. The interviews were conducted by the lead researcher and had prior experience conducting qualitative interviews in previous research projects. No relationship was established with participants prior to the study. Participants were given a full explanation of the study’s purpose before providing informed consent. Based on the research questions and guided by the study’s three theoretical frameworks, Self-Determination Theory [22,23], Social Support Theory [24,25], and Help-Seeking Behaviour Theory [7,26], the interview protocol was then developed. The draft protocol of the interview guide was subjected to expert review which comprised three experts with years of counselling experience and qualitative research. Revisions to the wording, sequence, and clarity of the items were based on the experts’ feedback. The revised protocol was then pilot-tested to expose the preliminary interview guide to critique and scrutiny to see if changes were needed [41]. The pilot test was conducted with two students and two counsellors who were then excluded from the final sample. Based on the pilot interviews, two refinements were made to the protocol. Firstly, the target interview duration was shortened, as some pilot participants began to feel uncomfortable in interviews extending beyond 30 minutes. Secondly, questions that proved ambiguous or required repeated clarification were restructured to convey their intended meaning more clearly. The overall interview flow and question sequence remained unchanged. The full interview guide, comprising the student and counsellor question sets organised by research question, is provided as Supplementary Material. For the main study, written informed consent was obtained from all participants for their participation and for the use of their data for research purposes. Semi-structured interviews were then conducted individually with students who used counselling services and counsellors. This allowed participants to share rich descriptions of phenomena while leaving the interpretation or analysis to the investigators [42]. Participants were asked about their experiences with mental health support services, barriers to service utilisation and delivery, and suggestions for improving these services. They were also encouraged to provide examples and describe specific incidents to enrich the data. All interviews were conducted face to face in a private setting to ensure confidentiality and participant comfort, with only the participant and interviewer present. Each participant was interviewed once, with no repeat interviews conducted. Brief field notes were taken during and immediately after each interview to supplement the audio recordings. Each interview lasted between 20 and 35 minutes. Participation was entirely voluntary, and participants were informed that they could withdraw at any time without penalty. No incentives were provided.

### 3.4. Data Analysis

This study combined deductive and inductive analytic strategies [43]. Deductive coding was guided by the research questions and by the core constructs of the study’s three selected theories which were: autonomy, competence, and relatedness dimensions (Self-Determination Theory); emotional, instrumental, informational, and appraisal dimensions (Social Support Theory); and problem-recognition, help-seeking attitudes, perceived stigma, and service-accessibility constructs (Help-Seeking Behaviour Theory). The inductive component enabled additional data-informed codes and patterns to be generated from participants’ accounts rather than being restricted to the predetermined theoretical constructs.

Drawing on Braun and Clarke’s (2006) six-phase framework [44], a hybrid deductive–inductive thematic analysis was conducted. Although the phases are described sequentially, the analysis was recursive, involving movement between coding, theme development, theme review and interpretation. The first phase, familiarisation with the data, involved transcribing all 25 interviews (15 students who used counselling services and 10 counsellors) verbatim. Both researchers then read and re-read the transcripts and recorded initial analytic observations to develop a detailed understanding of how the two participant groups described their experiences of mental health support services. During the second phase, initial codes were generated with the support of ATLAS.ti Version 24, which was used to organise and manage the data. Two PhD-qualified researchers coded the dataset independently using a combination of theory-informed and data-informed coding. Theory-informed coding was guided by dimensions drawn from the study’s three theoretical frameworks: autonomy, competence and relatedness from Self-Determination Theory; emotional, instrumental, informational and appraisal support from Social Support Theory; and problem recognition, attitudes, stigma and service accessibility from Help-Seeking Behaviour Theory. Data-informed coding enabled the researchers to generate additional codes from participants’ accounts. These included several counsellor-derived codes, such as staffing constraints and institutional undervaluation, that were not adequately captured by the three theoretical frameworks. The researchers subsequently compared their coding and discussed differences to refine the code definitions and develop a shared analytic interpretation. During the third phase, related codes were collated and used to develop candidate sub-themes across the combined student and counsellor dataset. This process included a systematic comparison of patterns within and between the two participant groups, particularly areas in which their accounts converged, diverged or reflected group-specific perspectives. During the fourth phase, the candidate sub-themes were reviewed against both the coded extracts and the complete dataset. Through an iterative process of returning to the original transcripts, the researchers refined the boundaries and content of the sub-themes and organised them within three overarching thematic domains: Experiences of Mental Health Support Services, Barriers and Challenges, and Strategies for Enhancement. This process also ensured that similarities and differences between students’ and counsellors’ accounts were represented accurately. During the fifth phase, each thematic domain and sub-theme was clearly defined and named. As an additional comparative analytic step, each subtheme was classified according to whether it reflected convergence, divergence or a perspective specific to one participant group. Codes that were not captured by the three theoretical frameworks were retained rather than being forced into the pre-existing theoretical categories, thereby helping to identify potential boundaries of the frameworks. During the sixth phase, the thematic findings were written up using illustrative participant extracts. The final thematic domains and sub-themes are presented in Section 4, while Section 5 interprets the findings in relation to the three guiding theoretical frameworks and the Malaysian higher education context.

### 3.5. Trustworthiness and Ethical Considerations

Several measures were taken to enhance the trustworthiness of the study. Credibility was supported through participant transcript checking, whereby participants were invited to review their interview transcripts and provide corrections or comments. Credibility was also strengthened through the researchers’ repeated engagement with the interview data and ongoing discussions about the developing analysis. Dependability was supported by maintaining a clear audit trail of data collection, coding, and theme development. Confirmability was enhanced through the audit trail, research-team discussions and the maintenance of reflexive notes throughout the study. These notes enabled the researchers to critically examine how their assumptions, experiences and theoretical positions may have influenced data collection and interpretation. Transferability was supported by providing detailed contextual descriptions of the participants, research setting and findings, allowing readers to assess the potential relevance of the findings to other contexts. These procedures reflected the principles of credibility, dependability, confirmability and transferability commonly used to evaluate trustworthiness in qualitative research. Measures were also taken to protect participant confidentiality and secure the research data. Identifying information was removed from the transcripts, and participant codes were used in the presentation of the findings. Student participants were identified using codes SP1 to SP15, while counsellors were identified using codes C1 to C10. All interview recordings, transcripts, and research documents were stored securely and were accessible only to the research team. The data were used solely for academic research purposes.

## 4. Analysis

### 4.1. Experiences of Mental Health Support Services

The first theme to emerge from the analysis was Experiences of Mental Health Support Services, comprising four sub-themes: Seeking Help and Trusting, Safe Relationships; Professional and Emotional Support; Understanding and Assessing Student Needs; and Facilitating Recovery and Growth. The first two sub-themes reflect areas of convergence between students and counsellors, while the latter two are counsellor-specific dimensions not directly voiced by students, offering a dyadic view of how support is experienced from both sides of the relationship.

The first sub-theme, Seeking Help and Trusting, Safe Relationships, emerged from the student codes Seeking Support and Safe Space for Mental Health Support, together with the counsellor code Active Listening and Creating a Safe Space.

“*I visited the university Counsellor to resolve my panic attack issue.*”

(SP2)

“*I approached the counselling unit when my stress began affecting my academic performance.*”

(SP6)

“*The campus Counsellor helped me learn to manage my anxiety during our meeting.*”

(SP4)

“*The counselling sessions provided a confidential environment where I could openly discuss my concerns.*”

(SP8)

Students consistently described seeking help once distress began affecting daily functioning, and emphasised trust and confidentiality as central to a positive experience. Counsellors independently described the same relational foundation from their side:

“*I listen attentively to their problems and suggest coping mechanisms once I know the cause of the problem. I take them to a safe and comfortable place to keep them at ease and allow them to speak about what is troubling them.*”

(C8)

“*I treat them with empathy and listen attentively to help them once I know what they are dealing with. I need to be patient, as this may take several sessions before I can zero in on the main problem. I help them cope first.*”

(C6)

This convergence is notable as both students and counsellors independently identified trust, safety, and active listening as foundational to the counselling relationship, aligning with the relatedness dimension of Self-Determination Theory.

The second sub-theme, Professional and Emotional Support, emerged from the student codes Mental Health Assistance and Counsellor Support, alongside the counsellor code Emotional Guidance.

“*I feel support services such as Counsellors are very helpful in overcoming mental health problems.*”

(SP3)

“*My mental health support came from both university Counsellors and trusted lecturers.*”

(SP5)

“*As a Counsellor, I assist people in managing their conflict or problems.*”

(C1)

“*I provide them with emotional guidance to help them self-manage their problems and self-regulate their emotions.*”

(C4)

“*I provide them with ways to handle their emotions. I understand the challenges these students face today. Some come in as an emotional wreck, shouting and crying after failing exams or a viva. They need to know how to handle their emotions, and that is what I do.*”

(C9)

Students identified counsellors and trusted staff as key sources of professional support, while counsellors described their role in almost identical terms, providing emotional guidance and helping students regulate difficult emotions. This is a second clear point of convergence between the two groups.

The third sub-theme, Understanding and Assessing Student Needs, was visible only on the counsellor side, comprising the codes Problem Identification and Severity Assessment and Understanding the Individual Before the Problem.

“*I assist students in identifying the root cause of the problem, what issue they are facing, and help them classify whether it is a severe or manageable problem.*”

(C7)

“*I help them by first surfacing their problem. Like if you cannot see the main reason or cause, you cannot be effective in solving their problems.*”

(C3)

“*I assess how serious the problem is. Not all cases need in depth counselling, as some can be issues that are solved easily. So, I first assess how serious the case is before proceeding.*”

(C6)

“*I help them first by understanding who they are, and only then study the problem. You must know what type of student you are dealing with before you can be effective in helping them.*”

(C5)

This sub-theme reflects a distinctly counsellor side dimension of the experience: the diagnostic and relational groundwork that precedes visible support, which students understandably did not describe from their own viewpoint. This divergence highlights a hidden layer of professional judgement that shapes support delivery but remains invisible to service users.

The fourth sub-theme, Facilitating Recovery and Growth, also emerged solely from counsellor accounts, through the code Supporting Self-Recovery.

“*I assist the client in passing through difficult phases in life and help the client to self-recover.*”

(C2)

“*I keep providing them with support wherever it can assist. To me I see what they need and assist them. It is a tough job, but I keep calm and keep going in supporting them.*”

(C3)

“*I help them recover from the shocks they have faced. I help them slowly and gradually overcome these shocks, cope with them, and recover.*”

(C10)

While students described the support they received, they did not explicitly frame their experience as a “recovery process”. This framing emerged distinctly from counsellors, who saw their role as extending beyond the immediate session toward longer-term resilience building.

In combination, these four sub-themes show that students’ and counsellors’ accounts of Experiences of Mental Health Support Services converge strongly around trust, safety, and emotional support, while diverging in the largely invisible diagnostic and recovery-facilitation work those counsellors carry out. Table 2 presents the theme development matrix.

### 4.2. Barriers and Challenges in Mental Health Support Services

The second theme, Barriers and Challenges in Mental Health Support Services, comprised four sub-themes: Awareness and Visibility of Services; Stigma and Institutional Attitudes; Staffing and Resource Constraints; and Professional Development and Process Gaps. Unlike Theme 1, where convergence was strong, this theme reveals a more divergent outcome to a certain level. While students and counsellors converge on issues of visibility and stigma, counsellors alone surfaced a set of structural and institutional constraints that shape service delivery from behind the scenes.

The first sub-theme, Awareness and Visibility of Services, emerged from the student code Lack of Services and the counsellor code Poor Visibility and Accessibility (Physical and Digital).

“*Most students are unaware of the mental health services available on campus.*”

(SP4)

“*The counselling services are not promoted frequently enough.*”

(SP11)

Counsellors independently identified the same root barrier, extending it to both physical and digital visibility:

“*We are tucked away in some “remoted” place as no one is aware of our services. I wonder how students will know how to access us.*”

(C4)

“*We are hidden in some part of the university. You know it never in a strategic location. Students generally do not know we exist and academic staff do not even know who we are.*”

(C5)

“*Why hide us somewhere where no one knows us, far away from everyone else? How are students going to find us? At least a prominent place on the website, or an e-counselling section on the university website.*”

(C2)

“*Institutions should give our mental health support division more importance. Otherwise, no one will know we exist to help students. Tell about us to students that we are here to help them.*”

(C8)

“*Students don’t know we are actually here to assist them. I think we need some promotion or marketing from the higher authority, or else we remain isolated and non-accessible.*”

(C9)

This is a genuine point of convergence: both students and counsellors independently identified the same barrier, that mental health services exist but are not well known or well promoted, though counsellors additionally highlighted a digital dimension not raised by students.

The second sub-theme, Stigma and Institutional Attitudes, emerged from the student code Acceptance Issues and the counsellor code Institutional Undervaluation.

“*Not many students accept mental health issues as a critical problem.*”

(SP5)

“*Some students prefer to keep mental health problems private.*”

(SP12)

Counsellors described a parallel, though structurally different, form of dismissal, one arising from the institution itself rather than from peers:

“*The emphasis given by management on our services is more about complying with government regulations. This neglect will hinder our progress and downplay our role in providing effective services.*”

(C3)

While students’ accounts reflect personal and peer level stigma, counsellors’ accounts reflect institutional level stigma, in the form of limited organisational commitment beyond regulatory compliance. This represents a partial convergence: both groups describe stigma as a barrier, but operating at different levels of the system.

The third sub-theme, Staffing and Resource Constraints, was raised exclusively by counsellors, through the code Insufficient Staffing Ratio and Workload Strain.

“*There is a lack of manpower given the ratio to the number of students in a university. We are only a two person unit expected to service a student population of 10,000 students! How do we deliver effective service?*”

(C3)

“*Lack of manpower to support the growing number of students seeking counselling help. We cannot support all these students and have enough time for them.*”

(C4)

“*We can operate running on three people. Imagine when a turnover takes place, we are so short handed. What happens to the students who come in desperately looking for help?*”

(C1)

“*The workload is demanding for more man workforce, and this issue is growing, especially with digital demands on students. Come on we need more people!.*”

(C6)

This barrier was invisible in student accounts, understandably so, since staffing ratios are an internal operational matter. It represents a clear divergence: a structural constraint that shapes service delivery capacity but falls entirely outside students’ knowledge and viewpoints.

The fourth sub-theme, Professional Development and Process Gaps, also emerged solely from counsellor accounts, through the codes Training and Development Gaps and Unclear Referral and Response Pathways.

“*We need more updated training to keep abreast of new developments if we are to support students.*”

(C1)

“*Give us more training, upskill us, equip us so we know how to handle the students... training must be put in place. No point having counsellors with short of new skills.*”

(C2)

“*We are not well-equipped to support students if don’t have new skill and knowledge. There are many new developments in mental health, but we are in the dark.*”

(C5)

“*To be more effective, we need a clearer pathway. If an academic lecturer raises a red flag, what are the next steps? After we diagnose, what are the next steps? This procedure should be clear to all staff.*”

(C2)

“*We should have a clear system and inform all staff how problematic and mentally afflicted students can be referred. A flow process shared with and taught to all staff, or else no one will know how student issues can be referred through the proper channels.*”

(C10)

Like the previous sub-theme, this reflects internal capacity and process constraints (training gaps and unclear referral procedures) that students would have no basis to observe or report.

Overall, this theme shows that while students and counsellors converge in identifying visibility and stigma as barriers, the counsellor side data reveals a second, largely invisible layer of structural barriers (staffing shortages, training gaps, and unclear referral pathways) that do not correspond to any student side code. Three of the counsellors derived codes in this theme (Insufficient Staffing Ratio and Workload Strain, Institutional Undervaluation, and Unclear Referral and Response Pathways) did not align clearly with any of the study’s three guiding theories, since Self-Determination Theory, Social Support Theory, and Help-Seeking Behaviour Theory are oriented toward individual and relational experience rather than institutional or structural conditions. This is addressed further as a limitation in the Discussion. Table 3 presents the theme development matrix.

### 4.3. Strategies for Enhancing Mental Health Support Services

The third theme, Strategies for Enhancing Mental Health Support Services, comprised five sub-themes: Assessment and Early Detection; Awareness, Stigma Reduction, and Digital Accessibility; Resilience and Coping Capacity; Stakeholder and Community Collaboration; and Referral and Response Pathways. As with the previous themes, students and counsellors converged strongly on some strategies while diverging on others that reflect counsellors’ distinct professional viewpoints.

The first sub-theme, Assessment and Early Detection, emerged from the student codes Mental Health Assessment and Training for Early Detection, together with the counsellor codes Mental Health Assessment (DASS21 Screening) and Early Stage Screening.

“*Surveys and questionnaires can be conducted to assess student mental health needs.*”

(SP1)

“*Training lecturers and student leaders to identify early signs of distress is important.*”

(SP5)

“*Basic mental support is performed using DASS21 screening.*”

(C1)

“*We need more early assessments on students to nip the problem in the bud as some clues can be detected. Compulsory early stage screening can detect potential cases before they get blown up.*”

(C4)

“*We need quicker detection and assessment before these problems explode out of control.*”

(C4)

Similar views on the value of early-stage screening were also echoed by C3. Both students and counsellors independently converged on the need for structured, proactive assessment tools rather than waiting for students to self-present in crisis.

The second sub-theme, Awareness, Stigma Reduction, and Digital Accessibility, emerged from the student codes Stigma Reduction and 24/7 Digital Support, together with the counsellor codes Psychoeducation and Awareness Programmes.

“*Run stigma reduction campaigns and peer support groups.*”

(SP3)

“*Awareness campaigns would encourage more students to seek help.*”

(SP13)

“*Digital counselling platforms should be available outside office hours.*”

(SP14)

“*We offer psychoeducation and healing techniques.*”

(C1)

“*Awareness initiatives help normalize help seeking behaviour among students.*”

(C10)

“*We need to have more talks and awareness campaigns on campus. It should be something we do regularly, maybe every month, not just once a semester, if we really want to make a difference.*”

(C5)

“*The university should have weekly online and digital posters targeting mental health issues, as this will give more awareness, since students are always on their mobile.*”

(C7)

“*Promote us more and allow us to give more talks to surface this mental health issue. The more openly we talk about it, the more we can reduce the stigma and help students become aware of the support available.*”

(C8)

Similar views on awareness building were also echoed by C3. This sub-theme shows clear convergence: students called for stigma reduction campaigns and after-hours digital access, while counsellors independently proposed recurring awareness talks and, notably, a digital or mobile-based promotional channel that closely reflected students’ calls for greater digital accessibility.

The third sub-theme, Resilience and Coping Capacity, emerged solely from the counsellor code Resilience Building.

“*We encourage students to face challenges and gradually develop resilience.*”

(C2)

“*I teach students how to face their challenges, understand the problem and help them to overcome it. Believe me or not, every problem is unique and has to be handled differently.*”

(C3)

“*I help students manage by identifying the problem and then taking the necessary actions. If you ask me, identification is the key focus in my approach.*”

(C5)

“*We always listen to students you know. You need to hear them out and then slowly help them to manage with their problems no matter how difficult the situation it is*.”

(C7)

This strategy was less prominent among students, reflecting a distinctly professional perspective: multiple counsellors (C2, C3, C5, C7) independently emphasised building students’ capacity to face and work through challenges—variously framed as fostering resilience, guided problem assessment, or sustained listening-based support—as a strategy extending beyond immediate crisis response. Students’ proposed strategies, by contrast, centred more on access and awareness.

The fourth sub-theme, Stakeholder and Community Collaboration, emerged from the student code Social Support Networks and the counsellor codes Collaboration with Stakeholders, Stakeholder Engagement, and Inter-University and Parental Collaboration.

“*Peer support groups can help students feel less isolated.*”

(SP15)

“*We collaborate with faculty and administrative departments to create awareness programs.*”

(C1)

“*We work with faculty members, parents, and residential colleges to help students.*”

(C2)

“*We need to work closely with all the relevant stakeholders and regulatory bodies. This can be within and outside the university. If we want to address mental health issues properly, everyone needs to work together, right?.*”

(C9)

“*We getting understand and work closely with everyone involved, including parents and other universities. Many of these issues affect students across Malaysia. But what I see at the moment is that each university have a practice of to deal with them on its own.*”

(C4)

Similar views on stakeholder collaboration were also echoed by C6. This is a case of partial convergence: students emphasised peer-to-peer support networks, while counsellors emphasised institutional, parental, and inter-university collaboration. Both point toward the same underlying principle of broadening the support network beyond the individual counselling session, but at different levels of the system.

The fifth sub-theme, Referral and Response Pathways, emerged solely from the counsellor code Psychiatric Referral.

“*Students who may harm themselves should be referred to psychiatrists.*”

(C2)

“*I refer a student to a psychiatrist when it is beyond my control to handle the student and the student has shown no improvement.*”

(C1)

“*We send the student for further clinical service when all our methods have failed and he or she requires advanced support.*”

(C8)

“*I try my best to help them recover and after all I have done, if I see no change, the student must be sent to external counselling and help.*”

(C9)

This pathway was consistently described across counsellors (C1, C2, C8, C9) as a last-resort escalation route rather than a routine strategy, triggered specifically when a case fell outside the counsellor’s own capacity to manage—whether due to risk of self-harm, absence of improvement despite sustained effort, or the limits of the counsellor’s own methods. This reflects a formal clinical pathway that sits entirely within the counsellor’s professional working scope, and one that students would not typically initiate or describe from their own perspective.

Hence, this theme shows strong convergence around assessment, awareness, and digital accessibility, partial convergence around collaboration, and clear divergence around resilience building and clinical referral, both of which are counsellor-driven professional strategies operating beyond students’ immediate view. Table 4 presents the theme development matrix.

## 5. Discussion

### 5.1. Convergence and Divergence Between Student and Counsellor Perspectives

The central contribution of this study’s dyadic design lies not in the individual themes but in the pattern of convergence and divergence between the two groups. Firstly, students and counsellors converged most strongly on the relational core of counselling (trust, confidentiality, and emotional support) and on the most visible service level problems: poor awareness, stigma, and the need for early assessment and outreach. This convergence strengthens the findings: when service users and providers independently identify the same relational foundations and the same barriers, those findings carry greater evidential weight than either perspective alone, and they identify the areas where intervention is least contested.

Secondly, the divergences reflected the two groups’ different viewpoints rather than genuine contradiction. Counsellors surfaced an entire layer of the support system that students could not see: understaffing, unclear referral procedures, training gaps, and weak institutional commitment. Students, conversely, emphasised what counsellors tended not to problematise: how services look, feel, and become known from the outside. Each account was accurate but incomplete on its own. This suggests that single-perspective studies of university mental health services, the dominant design in the existing literature [5,6] systematically miss one half of the service reality: student-only studies cannot detect structural constraints on delivery, while provider-only studies cannot detect experiential barriers to access.

Thirdly, several student and counsellor findings mirrored each other closely. Students reported not knowing services existed; counsellors reported being physically and digitally hidden. Students also described reactive, crisis-driven help-seeking, which is consistent with previous evidence that university students may perceive treatment as unnecessary [45]. In contrast, counsellors independently called for compulsory early screening to identify and address difficulties before they escalate. Together, these paired findings suggest that both groups were describing the same systemic shortcomings from different positions, indicating that the problems are structural rather than merely perceptual.

### 5.2. Theoretical Implications

The three guiding theories performed complementary rather than overlapping explanatory work, and their combined use maps onto the help-seeking journey as a sequence. Help-Seeking Behaviour Theory best explained whether support is sought at all: problem recognition, stigma, and service accessibility accounted for the largest share of coded findings across both groups [7,26]. Self-Determination Theory best explained why counselling works once accessed: the relatedness dimension underpinned the trust and safety students valued, while the competence dimension underpinned the coping capacity counsellors sought to build [22,23]. Social Support Theory best explained how support is sustained and extended beyond the counselling room, through lecturers, peers, families, and inter-institutional networks [24,25]. No single theory covers this whole sequence, which is why the integrated framework was needed.

The framework also showed clear limits. Three counsellor-derived codes (insufficient staffing ratios, institutional undervaluation, and unclear referral pathways) could not be credibly assigned to any of the three theories, all of which operate at the individual and relational level. These codes describe organisational and resource conditions. Their emergence, noted as a boundary in Section 4.2, indicates that psychological theories of help-seeking and motivation are necessary but not sufficient for understanding university mental health provision: a complete account requires an organisational level lens alongside them. This is a substantive theoretical implication of the dyadic design, since these codes surfaced only because providers were included in the sample.

### 5.3. The Malaysian Context

Several findings take on additional meaning in the Malaysian setting. The stigma students described (treating mental health as a private matter and doubting its legitimacy as a “critical problem”) is consistent with evidence that demographic and cultural factors, including ethnicity, religion, and age, shape stigma and help-seeking attitudes among Malaysians [4]. In collectivist settings, disclosure of psychological difficulty can implicate family reputation and not merely individual privacy, which may partly explain the strongly reactive help-seeking pattern observed: support is sought only once academic functioning, a socially legitimate concern, is visibly affected.

The counsellor-side findings also reflect structural features of Malaysian higher education. Counselling units are often small, centralised departments whose establishment may partly serve to fulfil regulatory requirements. This is illustrated by C3’s observation that management engagement was driven by the need to “comply with government regulations.” The reported ratio of two counsellors to approximately 10,000 students falls far short of international staffing benchmarks and gives concrete, local substance to what is often discussed only abstractly as resource constraints.

Situating the findings this way responds to the risk of treating help-seeking barriers as universal when their intensity and mechanics are in fact context-specific, a pattern suggested by comparing findings across different national settings. For example, stigma and help-seeking attitudes are shaped by ethnicity, religion, and age among Malaysian university students [4]. In contrast, a study in the UK found that more than one third of UK young adults experiencing mental health difficulties did not seek any help, with stigma, difficulty expressing concerns, cost, and a preference for self-reliance identified as the main barriers [46].

### 5.4. Practical Implications

Because the strategic themes incorporated both student and counsellor perspectives, the findings support recommendations that are more specific and contextually grounded than the generic calls for “awareness” and “access” commonly found in the literature. Firstly, universities could review counsellor staffing against student population ratios, since the workloads described here constrain every other improvement. Secondly, visibility requires both physical and digital remediation: relocating or signposting counselling units in central campus locations, and establishing a prominent website presence with an e-counselling entry point, as counsellors themselves proposed. Any expansion of 24/7 digital or technology-enabled services would also require careful consideration of platform infrastructure, financial sustainability, data privacy, cybersecurity safeguards, and institutional capacity. These represent important implementation requirements that Malaysian universities would need to assess from the outset. Thirdly, referral procedures should be formalised through a clearly documented pathway. This could include a flowchart outlining the steps academic staff should take when they identify a student experiencing difficulties, as well as the actions that follow a counsellor’s assessment. The pathway should be disseminated to all relevant staff to address the procedural gaps identified by counsellors. Fourthly, awareness initiatives could shift from one-off events to a recurring schedule, with counsellors specifically recommending monthly rather than once-per-semester activities. These efforts could be complemented by routine early screening using instruments already applied in local settings, such as the DASS-21. Lastly, structured continuing professional development would address the training gaps counsellors reported.

Given the qualitative and cross-sectional nature of this study, these recommendations are presented as evidence-informed starting points rather than established interventions. Their implementation and effectiveness should therefore be evaluated in future research.

## 6. Conclusions

This study addressed a gap in the current mental health literature through a qualitative dyadic design. By examining both the students’ and counsellors’ perspectives of mental health support services, this study has added novelty to the current literature. The design captured accounts of 15 students who used counselling services and 10 university counsellors, which resulted in a richer understanding of how mental health support services are experienced and delivered in Malaysian universities.

Based on the findings through a deductive and inductive process, three key themes emerged. The first theme was Experiences of Mental Health Support Services. It was observed that students only sought counselling support after their mental health distress affected their academic, personal and daily functioning. This reflects a reactive rather than preventive pattern of help-seeking amongst Malaysian university students. The findings further showed that during these encounters, students valued their confidentiality and psychological safety to possibly avoid any stigma. Counsellors described the same relational foundation of trust and safety, a clear point of convergence, but additionally articulated dimensions of their work that students did not observe: assessing student needs, building resilience, and supporting longer-term self-recovery.

The next theme which developed was Barriers and Challenges in Mental Health Support Services. Students identified limited awareness and persistent stigma as the key obstacles, being generally unaware that support services existed despite their availability. Counsellors converged on the visibility problem but additionally surfaced structural barriers largely invisible to students: insufficient staffing relative to student numbers, limited institutional commitment, training gaps, and unclear referral procedures.

The final theme centred on strategies towards improving mental health support services. In this theme, there was convergence from both students and counsellors on several recommendations. Amongst the most frequently noted were routine mental health screening, stigma reduction campaigns, early detection training for staff, peer support structures, and expanded digital and after-hours counselling. Counsellor-specific strategies further included stakeholder and inter-university collaboration and clear psychiatric referral pathways for higher-risk cases.

The three theories guiding this study worked complementarily to provide a strong explanatory reasoning for these findings. Self-Determination Theory provided theoretical arguments as to why students engaged with support services. Subsequently, Social Support Theory explained why students sustained that engagement. Help-Seeking Behaviour Theory explained what determined whether students sought help. The dyadic design proved most valuable in revealing where student expectations and counsellor practice aligned and where they diverged. Both groups converged on concerns relating to service visibility, stigma, and the need for early assessment. In contrast, structural constraints affecting service delivery—such as staffing, training, institutional commitment, and referral procedures—were identified primarily by counsellors, as were the need for resilience-building initiatives and formal psychiatric referral pathways.

This study extends these three theories to an under-researched context, Malaysian higher education. It also demonstrates the value of pairing student and counsellor data within one qualitative design. For mental health practice, the findings give university administrators a clear basis for action. Universities should sustain awareness campaigns rather than run them as one-off events. Stigma reduction programmes should be prioritised. Digital and peer-based access points should be expanded. Mental health support functions best as a coordinated system. Its effectiveness depends on alignment between student needs, counsellor capacity, and institutional visibility.

## 7. Limitations and Future Research Recommendation

Several limitations are acknowledged in this study and subsequent recommendations are provided.

- Methodological Limitation: The qualitative dyadic design had a relatively small sample size (15 students, 10 counsellors). This limits the generalisability of the findings, as they reflect the experiences of these individuals rather than patterns that hold across the broader population of Malaysian universities. Moving forward, future research could broaden the sample size to capture variation across university type (public vs. private), region, discipline, or severity of mental health issue. In addition, comparative studies involving universities from different countries or cultural settings may help identify context-specific factors influencing mental health support utilisation. Future studies could also triangulate interview-based accounts with objective institutional indicators, such as counsellor to student ratios, service utilisation rates, waiting times, and mental health budget allocations, to complement self-reported data with organisational evidence.
- Sample eligibility limitation: This study’s eligibility criteria required that student participants had already used university counselling services at least once. As a result, the perspectives, barriers, and unmet needs of students who did not seek or use mental health support services are not represented in this study. This is a significant limitation for a study examining help-seeking behaviour and service accessibility specifically, since the barriers that prevent help-seeking in the first place may differ substantially from the barriers experienced by those who have already accessed support. Future research should include students who have not sought counselling support, to directly capture the perspectives of non-help seekers and identify barriers operating earlier in the help-seeking pathway.
- Contextual and temporal limitations*:* The current study findings are specific to the Malaysian university context, which limits their generalisability elsewhere. In addition, the use of a cross-sectional design captures a single time period and does not track changes across a student’s academic journey. Future studies should expand the scope of this research by including participants from different regions or continents covering a larger number of public and private universities. In addition, the use of a longitudinal research design will provide a more comprehensive study across a student’s entire tertiary academic journey. Such studies would provide deeper insights into the long-term effectiveness of university mental health support services and intervention programmes.

## Figures and Tables

**Table 1:** Demographic profile of participants.

Characteristic	Students Who Used Counselling Services (*n* = 15)	Counsellors (*n* = 10)	Total (*N* = 25)
Gender			
Male	8 (53.3%)	5 (50.0%)	13 (52.0%)
Female	7 (46.7%)	5 (50.0%)	12 (48.0%)
Age Range (years)	20–23	–	–
Participant Type			
Undergraduate Students	15 (100.0%)	–	15 (60.0%)
Registered Counsellors	–	8 (80.0%)	8 (32.0%)
Senior Counsellors	–	2 (20.0%)	2 (8.0%)
Year of Study			
Year 1	3 (20.0%)	–	3 (12.0%)
Year 2	4 (26.7%)	–	4 (16.0%)
Year 3	4 (26.7%)	–	4 (16.0%)
Year 4	4 (26.7%)	–	4 (16.0%)
Professional Experience			
Counselling Experience	**–**	7–16 years	–

Note: *N* = 25. The study involved 15 students who have used counselling services and 10 counsellors who were actively involved in providing counselling services within Malaysian higher education institutions.

**Table 2:** Theme development matrix for Experiences of Mental Health Support Services.

Theme	Sub-Theme	Code	Supporting Participants	Theory Match	Dimension	Convergence/Divergence
Experiences of Mental Health Support Services	Seeking Help and Trusting, Safe Relationships	Seeking Support	SP2, SP6	Help-Seeking Behaviour Theory	Problem Recognition & Professional Help-Seeking	Convergent
Experiences of Mental Health Support Services	Seeking Help and Trusting, Safe Relationships	Safe Space for Mental Health Support	SP4, SP8	Self-Determination Theory	Relatedness	Convergent
Experiences of Mental Health Support Services	Seeking Help and Trusting, Safe Relationships	Active Listening and Creating a Safe Space	C6, C8	Self-Determination Theory	Relatedness	Convergent
Experiences of Mental Health Support Services	Professional and Emotional Support	Mental Health Assistance	SP3, SP5	Social Support Theory	Emotional Support	Convergent
Experiences of Mental Health Support Services	Professional and Emotional Support	Counsellor Support	SP3, SP5	Social Support Theory	Informational Support	Convergent
Experiences of Mental Health Support Services	Professional and Emotional Support	Emotional Guidance	C1, C4, C9	Social Support Theory	Emotional Support	Convergent
Experiences of Mental Health Support Services	Understanding and Assessing Student Needs	Problem Identification and Severity Assessment	C3, C6, C7	Help-Seeking Behaviour Theory	Problem Recognition (counsellor-side)	Divergent
Experiences of Mental Health Support Services	Understanding and Assessing Student Needs	Understanding the Individual Before the Problem	C5	Self-Determination Theory	Relatedness	Divergent
Experiences of Mental Health Support Services	Facilitating Recovery and Growth	Supporting Self Recovery	C2, C3, C10	Self-Determination Theory	Competence	Divergent

**Table 3:** Theme development matrix for Barriers and Challenges in Mental Health Support Services.

Theme	Sub-Theme	Code	Supporting Participants	Theory Match	Dimension	Convergence/Divergence
Barriers and Challenges in Mental Health Support Services	Awareness and Visibility of Services	Lack of Services	SP4, SP11	Help-Seeking Behaviour Theory	Service Accessibility	Convergent
Barriers and Challenges in Mental Health Support Services	Awareness and Visibility of Services	Poor Visibility and Accessibility (Physical and Digital)	C2, C4, C5, C8, C9	Help-Seeking Behaviour Theory	Service Accessibility	Convergent
Barriers and Challenges in Mental Health Support Services	Stigma and Institutional Attitudes	Acceptance Issues	SP5, SP12	Help-Seeking Behaviour Theory	Perceived Stigma	Partially Convergent
Barriers and Challenges in Mental Health Support Services	Stigma and Institutional Attitudes	Institutional Undervaluation	C3, C5, C8	(outside theoretical scope)	—	Partially Convergent
Barriers and Challenges in Mental Health Support Services	Staffing and Resource Constraints	Insufficient Staffing Ratio and Workload Strain	C1, C3, C4, C6, C7	(outside theoretical scope)	—	Divergent
Barriers and Challenges in Mental Health Support Services	Professional Development and Process Gaps	Training and Development Gaps	C1, C2, C5, C7	Self-Determination Theory	Competence	Divergent
Barriers and Challenges in Mental Health Support Services	Professional Development and Process Gaps	Unclear Referral and Response Pathways	C1, C2, C10	(outside theoretical scope)	—	Divergent

**Table 4:** Theme development matrix for Strategies for Enhancing Mental Health Support Services.

Theme	Sub-Theme	Code	Supporting Participants	Theory Match	Dimension	Convergence/Divergence
Strategies for Enhancing Mental Health Support Services	Assessment and Early Detection	Mental Health Assessment	SP1	Help-Seeking Behaviour Theory	Problem Recognition	Convergent
Strategies for Enhancing Mental Health Support Services	Assessment and Early Detection	Training for Early Detection	SP5	Help-Seeking Behaviour Theory	Early Intervention	Convergent
Strategies for Enhancing Mental Health Support Services	Assessment and Early Detection	Mental Health Assessment (DASS21 Screening)	C1	Help-Seeking Behaviour Theory	Problem Recognition	Convergent
Strategies for Enhancing Mental Health Support Services	Assessment and Early Detection	Early-Stage Screening	C3, C4	Help-Seeking Behaviour Theory	Problem Recognition	Convergent
Strategies for Enhancing Mental Health Support Services	Awareness, Stigma Reduction, and Digital Accessibility	Stigma Reduction	SP3, SP13	Help-Seeking Behaviour Theory	Attitudes Towards Help-Seeking	Convergent
Strategies for Enhancing Mental Health Support Services	Awareness, Stigma Reduction, and Digital Accessibility	24/7 Digital Support	SP14	Help-Seeking Behaviour Theory	Service Accessibility	Convergent
Strategies for Enhancing Mental Health Support Services	Awareness, Stigma Reduction, and Digital Accessibility	Psychoeducation	C1	Self-Determination Theory	Competence	Convergent
Strategies for Enhancing Mental Health Support Services	Awareness, Stigma Reduction, and Digital Accessibility	Awareness Programmes	C3, C5, C7, C8, C10	Help-Seeking Behaviour Theory	Mental Health Awareness	Convergent
Strategies for Enhancing Mental Health Support Services	Resilience and Coping Capacity	Resilience Building	C2, C3, C5, C7	Self-Determination Theory	Competence	Divergent
Strategies for Enhancing Mental Health Support Services	Stakeholder and Community Collaboration	Social Support Networks	SP15	Social Support Theory	Emotional Support	Partially Convergent
Strategies for Enhancing Mental Health Support Services	Stakeholder and Community Collaboration	Collaboration with Stakeholders	C1	Social Support Theory	Instrumental Support	Partially Convergent
Strategies for Enhancing Mental Health Support Services	Stakeholder and Community Collaboration	Stakeholder Engagement	C2, C6, C9	Social Support Theory	Instrumental Support	Partially Convergent
Strategies for Enhancing Mental Health Support Services	Stakeholder and Community Collaboration	Inter-University and Parental Collaboration	C4	Social Support Theory	Instrumental Support	Partially Convergent
Strategies for Enhancing Mental Health Support Services	Referral and Response Pathways	Psychiatric Referral	C1, C2, C8, C9	Help-Seeking Behaviour Theory	Formal Help-Seeking Pathway	Divergent

## Data Availability

The data supporting the findings of this study are included within the manuscript. Additional anonymized data may be made available from the corresponding author upon reasonable request, subject to ethical and privacy considerations.

## Supplementary Materials

The following supporting information can be downloaded at: https://www.mdpi.com/article/10.3390/healthcare14162606/s1, S1: Interview Guide; S2: COREQ Checklist.

## References

[1] United Nations (2024). United Nations System Mental Health and Well-Being Strategy for 2024 and Beyond. https://hr.un.org/sites/default/files/2025-10/UN_system_mental_health_and_well_being_strategy_for_2024.pdf.

[2] UNICEF Malaysia, Institute for Youth Research Malaysia (2023). Malaysian Youth Mental Health Index. https://www.unicef.org/malaysia/reports/malaysian-youth-mental-health-index-2023.

[3] Institute for Public Health (2020). National Health and Morbidity Survey (NHMS) 2019: Fact Sheet.

[4] Muda N., Ahmad Raphaie F.K. (2025). A multiple correspondence analysis towards understanding of public and self-stigma in mental health help-seeking among Malaysian university students. Sci. Rep..

[5] Campbell F., Blank L., Cantrell A., Baxter S., Blackmore C., Dixon J., Goyder E. (2022). Factors that influence mental health of university and college students in the UK: A systematic review. BMC Public Health.

[6] Eisenberg D., Hunt J., Speer N. (2012). Help Seeking for Mental Health on College Campuses: Review of Evidence and Next Steps for Research and Practice. Harv. Rev. Psychiatry.

[7] Gulliver A., Griffiths K.M., Christensen H. (2010). Perceived barriers and facilitators to mental health help-seeking in young people: A systematic review. BMC Psychiatry.

[8] Okunade B.A., Adediran F.E., Maduka C.P., Adegoke A.A. (2023). Community-Based Mental Health Interventions in Africa: A Review and Its Implications for U.S. Healthcare Practices. Int. Med. Sci. Res. J..

[9] McGorry P.D., Mei C., Chanen A., Hodges C., Alvarez-Jimenez M., Killackey E. (2022). Designing and scaling up integrated youth mental health care. World Psychiatry.

[10] Osborn T.L., Venturo-Conerly K.E., Wasil A.R., Rodriguez M., Roe E., Alemu R., Arango G.S., Gan J., Wasanga C., Schleider J.L. (2020). The Shamiri group intervention for adolescent anxiety and depression: Study protocol for a randomized controlled trial of a lay-provider-delivered, school-based intervention in Kenya. Trials.

[11] Elgar F.J., Pförtner T.-K., Rothwell D. (2024). Socioeconomic differences and global trends in youth wellbeing and emotional distress in 165 countries and territories. Health Place.

[12] Angotti M.A. (2025). Rethinking Youth Mental Health: Challenges, Interventions, and Cultural Perspectives. J. Future Soc. Educ..

[13] Harte P., Barry M.M. (2024). A scoping review of the implementation and cultural adaptation of school-based mental health promotion and prevention interventions in low-and middle-income countries. Camb. Prism. Glob. Ment. Health.

[14] Sheikh A., Jacob J., Vostanis P., Ruby F., Spuerck I., Stankovic M., Morgan N., Mota C.P., Ferreira R., Eruyar Ş. (2024). What Should Personalised Mental Health Support Involve? Views of Young People with Lived Experience and Professionals from Eight Countries. Adm. Policy Ment. Health Ment. Health Serv. Res..

[15] Kaku S.M., Sibeoni J., Basheer S., Chang J.P.-C., Dahanayake D.M.A., Irarrazaval M., Lachman J.M., Mapayi B.M., Mejia A., Orri M. (2022). Global child and adolescent mental health perspectives: Bringing change locally, while thinking globally. Child Adolesc. Psychiatry Ment. Health.

[16] Moitra M., Owens S., Hailemariam M., Wilson K.S., Mensa-Kwao A., Gonese G., Kamamia C.K., White B., Young D.M., Collins P.Y. (2023). Global Mental Health: Where We Are and Where We Are Going. Curr. Psychiatry Rep..

[17] (2024). Closing the global gap in adolescent mental health. Nat. Med..

[18] Uhlhaas P.J., Davey C.G., Mehta U.M., Shah J., Torous J., Allen N.B., Avenevoli S., Bella-Awusah T., Chanen A., Chen E.Y.H. (2023). Towards a youth mental health paradigm: A perspective and roadmap. Mol. Psychiatry.

[19] Marciano L., Vocaj E., Bekalu M.A., La Tona A., Rocchi G., Viswanath K. (2023). The Use of Mobile Assessments for Monitoring Mental Health in Youth: Umbrella Review. J. Med. Internet Res..

[20] Gruber J., Hinshaw S.P., Clark L.A., Rottenberg J., Prinstein M.J. (2023). Young Adult Mental Health Beyond the COVID-19 Era: Can Enlightened Policy Promote Long-Term Change?. Policy Insights Behav. Brain Sci..

[21] Cosma A., Black M., Vuckovic S., Pavic I., Fonseca H., Lazzerini M. (2025). The changing epidemiology of child and adolescent mental health requires an immediate policy response. Public Health Pract..

[22] Deci E.L., Ryan R.M. (1985). Intrinsic Motivation and Self-Determination in Human Behavior.

[23] Ryan R.M., Deci E.L. (2000). Self-determination theory and the facilitation of intrinsic motivation, social development, and well-being. Am. Psychol..

[24] Cohen S., Wills T.A. (1985). Stress, social support, and the buffering hypothesis. Psychol. Bull..

[25] House J.S. (1981). Work Stress and Social Support.

[26] Rickwood D., Deane F.P., Wilson C.J., Ciarrochi J. (2005). Young people’s help-seeking for mental health problems. Aust. E-J. Adv. Ment. Health.

[27] Creswell J.W., Creswell J.D. (2018). Qualitative, Quantitative, and Mixed Methods Approaches.

[28] Lincoln Y.S., Guba E.G. (1985). Naturalistic Inquiry.

[29] Saunders M., Lewis P., Thornhill A. (2019). Research Methods for Business Students.

[30] Yin R.K. (2013). Case Study Research: Design and Methods.

[31] Eisikovits Z., Koren C. (2010). Approaches to and Outcomes of Dyadic Interview Analysis. Qual. Health Res..

[32] Morgan D.L., Ataie J., Carder P., Hoffman K. (2013). Introducing Dyadic Interviews as a Method for Collecting Qualitative Data. Qual. Health Res..

[33] Caldwell K. (2014). Dyadic interviewing: A technique valuing interdependence in interviews with individuals with intellectual disabilities. Qual. Res..

[34] Kenny D.A., Kashy D.A., Cook W.L. (2006). Dyadic Data Analysis.

[35] Palinkas L.A., Horwitz S.M., Green C.A., Wisdom J.P., Duan N., Hoagwood K. (2015). Purposeful Sampling for Qualitative Data Collection and Analysis in Mixed Method Implementation Research. Adm. Policy Ment. Health Ment. Health Serv. Res..

[36] Patton M.Q. (2015). Qualitative Research & Evaluation Methods.

[37] Guest G., Bunce A., Johnson L. (2006). How Many Interviews Are Enough?: An Experiment with Data Saturation and Variability. Field Methods.

[38] Braun V., Clarke V. (2021). To saturate or not to saturate? Questioning data saturation as a useful concept for thematic analysis and sample-size rationales. Qual. Res. Sport Exerc. Health.

[39] Malterud K., Siersma V.D., Guassora A.D. (2016). Sample Size in Qualitative Interview Studies: Guided by Information Power. Qual. Health Res..

[40] Tong A., Sainsbury P., Craig J. (2007). Consolidated criteria for reporting qualitative research (COREQ): A 32-item checklist for interviews and focus groups. Int. J. Qual. Health Care.

[41] Kallio H., Pietilä A., Johnson M., Kangasniemi M. (2016). Systematic methodological review: Developing a framework for a qualitative semi-structured interview guide. J. Adv. Nurs..

[42] DiCicco-Bloom B., Crabtree B.F. (2006). The qualitative research interview. Med. Educ..

[43] Saldana J. (2025). The Coding Manual for Qualitative Researchers.

[44] Braun V., Clarke V. (2006). Using thematic analysis in psychology. Qual. Res. Psychol..

[45] Czyz E.K., Horwitz A.G., Eisenberg D., Kramer A., King C.A. (2013). Self-Reported Barriers to Professional Help Seeking Among College Students at Elevated Risk for Suicide. J. Am. Coll. Health.

[46] Salaheddin K., Mason B. (2016). Identifying barriers to mental health help-seeking among young adults in the UK: A cross-sectional survey. Br. J. Gen. Pract..

