# Supplementary material for: Mental Health Support Services in Universities: A Dyadic Exploration of Students’ and Counsellors’ Perspectives
Source: Healthcare (Basel). 2026 Aug 19;14(16):2606. doi: 10.3390/healthcare14162606 (PMC13512724; doi:10.3390/healthcare14162606)
Supplement: Supplementary file 1 [file healthcare-14-02606-s001.zip › Supplementary_S1_Interview_Guide.pdf]

Semi-Structured Interview Guide

This interview guide was used to conduct semi-structured interviews with student participants (SP1–SP15) and counsellors (C1–C10). Questions were developed based on the study's three research questions and its guiding theoretical framework (Self-Determination Theory, Social Support Theory, and Help-Seeking Behaviour Theory), and were refined through expert review and pilot testing as described in Section 3.3 of the manuscript.

Opening Question (asked of all participants)

Can you tell me a little about your experience with / role in university mental health support services?

RQ1: Experiences of Mental Health Support Services

**Student Questions**	**Counsellor Questions**
1. Can you tell me about your experience with university mental health support services?	1. Can you describe your role in supporting students' mental health at your university?
2. What led you to seek help from counselling services?	2. How do you create a safe and comfortable environment for students to open up?
3. How would you describe your experience during your counselling sessions?	3. How would you describe your approach to helping students manage personal difficulties?
4. Who else, if anyone, provided you with support during this time?	4. How do you go about identifying the root cause of a student's problem?
5. How helpful have counselling services been in addressing your concerns?	5. How do you get to know a student before addressing their specific issue?
	6. How do you support students in working through and recovering from difficult periods?

RQ2: Barriers and Challenges

**Student Questions**	**Counsellor Questions**
1. In your view, are students generally aware of the mental health services available on campus?	1. Do students and staff know where to find your services, both on campus and online?
2. How do you think students generally view or talk about mental health issues?	2. How would you describe the level of institutional support and priority given to counselling services?
	3. How manageable is your current caseload relative to your team's size?
	4. Do you feel adequately trained and up to date to handle the range of cases you see?
	5. How clear is the referral process when a lecturer or staff member flags a concerning student?

RQ3: Strategies for Enhancement

Student Questions	Counsellor Questions
1. What do you think universities could do to identify students who may need support earlier?	1. What tools or methods do you use to assess students' mental health needs?
2. What kinds of awareness or outreach efforts would encourage more students to seek help?	2. What kind of psychoeducation or preventive support do you offer students?
3. Are there ways counselling services could be made more accessible?	3. What awareness initiatives would help normalize help-seeking and reduce stigma?
4. What role could peer support play in helping students?	4. How do you help students build resilience and coping skills for the future?
	5. Which departments, stakeholders, or external parties do you collaborate with to support students?
	6. Do you see value in universities collaborating with each other or with parents on this issue?
	7. Under what circumstances would you refer a student to a psychiatrist or external clinical service?

Closing Question (asked of all participants)

Student version: Is there anything else about your experience with mental health support services that you would like to share?

Counsellor version: Is there anything else about your role or experience supporting student mental health that you would like to share?
