# Supplementary material for: Mental Health Support Services in Universities: A Dyadic Exploration of Students’ and Counsellors’ Perspectives
Source: Healthcare (Basel). 2026 Aug 19;14(16):2606. doi: 10.3390/healthcare14162606 (PMC13512724; doi:10.3390/healthcare14162606)
Supplement: Supplementary file 1 [file healthcare-14-02606-s001.zip › Supplementary_S2_COREQ_Checklist.pdf]

**COREQ (COnsolidated criteria for REporting Qualitative research) Checklist**

Completed for: Mental Health Support Services in Universities: A Dyadic Exploration of Students' and Counsellors' Perspectives (healthcare-4419926). Checklist based on Tong, A., Sainsbury, P., & Craig, J. (2007). Consolidated criteria for reporting qualitative research (COREQ): a 32-item checklist for interviews and focus groups. International Journal for Quality in Health Care, 19(6), 349–357.

| No. / Item | Guide Question / Description | Reported in |
|---|---|---|
| **Domain 1: Research team and reflexivity** | | |
| 1. Interviewer/facilitator | Which author/s conducted the interview or focus group? | Section 3.3 — interviews conducted by a PhD-qualified member of the research team |
| 2. Credentials | What were the researcher's credentials? E.g. PhD, MD | Section 3.3 — PhD |
| 3. Occupation | What was their occupation at the time of the study? | Section 3.3 — academic researcher (member of the research team) |
| 4. Gender | Was the researcher male or female? | Section 3.3 — male |
| 5. Experience and training | What experience or training did the researcher have? | Section 3.3 — prior experience conducting qualitative interviews from previous research projects |
| 6. Relationship established | Was a relationship established prior to study commencement? | Section 3.3 — no prior relationship with participants |
| 7. Participant knowledge of the interviewer | What did the participants know about the researcher? | Section 3.3 — participants received a full explanation of the study's purpose, including its theoretical framework, before consent |
| 8. Interviewer characteristics | What characteristics were reported about the interviewer/facilitator? | Sections 3.3 and 3.5 — reflexive notes maintained throughout to surface assumptions and minimise bias |
| **Domain 2: Study design** | | |
| 9. Methodological orientation and theory | What methodological orientation was stated to underpin the study? | Sections 3.1 and 3.4 — interpretivist philosophy; qualitative exploratory dyadic design; thematic analysis (Braun & Clarke, 2006) |
| 10. Sampling | How were participants selected? | Section 3.2 — purposive sampling |
| 11. Method of approach | How were participants approached? | Section 3.2 — email invitation |
| 12. Sample size | How many participants were in the study? | Section 3.2 and Table 1 — 15 students who used counselling services and 10 counsellors (N = 25) |
| 13. Non-participation | How many people refused to participate or dropped out? Reasons? | Section 3.2 — none; all approached individuals agreed to participate |
| 14. Setting of data collection | Where was the data collected? | Sections 3.2 and 3.3 — multiple universities across Malaysia; face-to-face in a private setting |
| 15. Presence of non-participants | Was anyone else present besides the participants and researchers? | Section 3.3 — only the participant and interviewer were present |

| | | |
|---|---|---|
| 16. Description of sample | What are the important characteristics of the sample? | Table 1 — gender, age range (students: 20–23 years), participant type, year of study, counselling experience (7–16 years); Section 3.2 — eligibility (prior use of counselling services; no formal diagnosis required) |
| 17. Interview guide | Were questions, prompts, guides provided by the authors? Was it pilot tested? | Section 3.3 and Supplementary File (Semi-Structured Interview Guide) — expert-reviewed and pilot tested with two students and two counsellors |
| 18. Repeat interviews | Were repeat interviews carried out? | Section 3.3 — no; single interview per participant |
| 19. Audio/visual recording | Did the research use audio or visual recording to collect the data? | Section 3.4 — all interviews audio recorded and transcribed verbatim |
| 20. Field notes | Were field notes made during and/or after the interview? | Section 3.3 — brief field notes taken during and/or immediately after each interview |
| 21. Duration | What was the duration of the interviews? | Section 3.3 — 20–35 minutes; duration justified by the focused semi-structured protocol and pilot timing |
| 22. Data saturation | Was data saturation discussed? | Section 3.2 — consistent with Braun & Clarke (2021), saturation was not used as the sample-size rationale; adequacy was assessed reflexively via information power (Malterud et al., 2016) |
| 23. Transcripts returned | Were transcripts returned to participants for comment and/or correction? | Section 3.5 — yes; transcripts shared with participants for review and comment |
| **Domain 3: Analysis and findings** | | |
| 24. Number of data coders | How many data coders coded the data? | Section 3.4 — two PhD-qualified researchers coded independently; discrepancies resolved by discussion to consensus |
| 25. Description of the coding tree | Did authors provide a description of the coding tree? | Section 3.4 and Tables 2–4 — theme development matrices (theme, sub-theme, code, participants, theory match) |
| 26. Derivation of themes | Were themes identified in advance or derived from the data? | Section 3.4 — combined inductive and deductive strategies; themes refined through iterative comparison with transcripts |
| 27. Software | What software, if applicable, was used to manage the data? | Section 3.4 — ATLAS.ti Version 24 |
| 28. Participant checking | Did participants provide feedback on the findings? | Section 3.5 — member checking via transcript review by participants |

| | | |
|---|---|---|
| 29. Quotations presented | Were participant quotations presented to illustrate the themes/findings? Was each quotation identified? | Section 4 — verbatim quotations throughout, identified by participant codes (SP1–SP15; C1–C10) |
| 30. Data and findings consistent | Was there consistency between the data presented and the findings? | Sections 4–5 and Tables 2–4 — findings anchored in quoted data and theme matrices |
| 31. Clarity of major themes | Were major themes clearly presented in the findings? | Section 4 — three major themes (Experiences; Barriers and Challenges; Strategies) |
| 32. Clarity of minor themes | Is there a description of diverse cases or discussion of minor themes? | Sections 4–5 — divergent and partially convergent findings explicitly labelled and interpreted; codes outside the theoretical framework identified |
